# A pilot study on anti‐reflux mucoplasty with valve as novel endoscopic therapy for gastroesophageal reflux disease

**DOI:** 10.1002/deo2.70131

**Published:** 2025-05-04

**Authors:** Kazuki Yamamoto, Haruhiro Inoue, Ippei Tanaka, Rei Miyake, Masachika Saino, Kei Ushikubo, Miyuki Iwasaki, Yohei Nishikawa, Satoshi Abiko, Boldbaatar Gantuya, Manabu Onimaru, Mayo Tanabe

**Affiliations:** ^1^ Digestive Diseases Center Showa University Koto Toyosu Hospital Tokyo Japan; ^2^ Department of Gastroenterology Mongolian National University of Medical Sciences Ulaanbaatar Mongolia; ^3^ Endoscopy Unit Mongolia Japan Hospital Ulaanbaatar Mongolia

**Keywords:** anti‐reflux mucosectomy, endoscopic submucosal dissection, gastroesophageal reflux disease, non‐erosive reflux disease, proton pump inhibitors

## Abstract

**Background and aims:**

Endoscopic anti‐reflux therapies like anti‐reflux mucosectomy (ARMS) and anti‐reflux mucosal ablation have shown efficacy for gastroesophageal reflux disease (GERD) in systematic reviews and meta‐analyses. Anti‐reflux mucoplasty (ARM‐P), a refinement of ARMS, incorporates immediate closure of the resection site to reduce complications. Recently, anti‐reflux mucosal valvuloplasty (ARMV), which employs endoscopic submucosal dissection to create a mucosal valve, was introduced but retains ARMS's limitations, requiring extensive incisions (three‐quarters to four‐fifths circumference). To address these challenges, we developed anti‐reflux mucoplasty with valve (ARM‐P/V), integrating ARMV's valvuloplasty with ARM‐P's closure technique to improve safety and reduce complications. This pilot study evaluates the safety, feasibility, and efficacy of ARM‐P/V.

**Methods:**

This retrospective study reviewed data from patients undergoing ARM‐P/V for proton pump inhibitor (PPI)‐refractory or PPI‐dependent GERD at Showa University Koto Toyosu Hospital, Tokyo, from April to August 2024. Symptom severity and quality of life were assessed using validated questionnaires (GERD‐Health Related Quality of Life Questionnaire [GERD‐HRQL], GERD Questionnaire [GerdQ], and Frequency Scale for the Symptoms of GERD [FSSG]), comparing pre‐ and post‐treatment scores. PPI discontinuation rates were also analyzed.

**Results:**

Eighteen patients (mean age 55.4 years) underwent ARM‐P/V. Within 3 months, 72.2% (13/18) reduced or discontinued PPI use. GERD‐HRQL scores improved from 20.3 to 10.9 (*p* = 0.004), GerdQ from 10.4 to 6.9 (*p* < 0.001), and FSSG from 24.0 to 13.2 (*p* < 0.001). No severe complications (Clavien‐Dindo Grade ≥3), delayed bleeding or dysphagia requiring balloon dilation were reported.

**Conclusions:**

ARM‐P/V demonstrates safety, technical feasibility, and short‐term efficacy in GERD treatment. As a refinement of ARMV, it offers a promising alternative to current techniques.

## INTRODUCTION

Gastroesophageal reflux disease (GERD) is a widespread chronic disorder of the upper digestive tract, with its incidence increasing worldwide.^1^ While acid‐suppressive medications provide symptom relief for many, nearly 40% of patients continue to suffer from reflux symptoms despite medical treatment.^2^ Surgical procedures like Nissen and Toupet fundoplications are well‐recognized as effective solutions for GERD.^3^, ^4^, ^5^ Nevertheless, the need for minimally invasive alternatives has driven the exploration of numerous endoscopic techniques.^6^, ^7^, ^8^, ^9^, ^10^, ^11^ However, a universally recognized standard for endoscopic GERD treatment has yet to emerge.

To address this gap, we have pioneered and demonstrated the benefits of endoscopic anti‐reflux therapy (EARTh) for GERD, including techniques such as anti‐reflux mucosectomy (ARMS),^12^, ^13^ anti‐reflux mucosal ablation (ARMA),^14^, ^15^ and anti‐reflux mucoplasty (ARM‐P).^16^, ^17^ ARMS involves circumferential mucosal resection of three‐quarters to four‐fifths of the cardia mucosa along the lesser curvature, while ARMA substitutes mucosal resection with mucosal ablation. ARM‐P, a refinement of ARMS, entails a more conservative approach, limiting resection to one‐third of the mucosa and incorporating immediate closure of the resection site. Among these treatments, ARMS has gained approval as an endoscopic GERD therapy covered by medical insurance in Japan. Long‐term studies affirm its effectiveness for up to 5 years,^18^ while ARMA has demonstrated sustained efficacy for up to 3 years, confirming their roles as reliable options for GERD management.^19^ Both techniques have been extensively studied, with systematic reviews and meta‐analyses underscoring their safety and effectiveness.^20^, ^21^, ^22^ ARM‐P offers additional potential advantages by reducing variability in cardio‐pasty outcomes and lowering the risk of delayed bleeding or transient strictures through its closure‐focused design.^16^

Lu J et al. recently published a technique called anti‐reflux mucosal valvuloplasty (ARMV), which involves applying endoscopic submucosal dissection to create a valve by preserving the mucosa without removing it.^23^ Despite its innovative approach, ARMV faces similar challenges as ARMS and ARMA, as it requires making an incision covering three‐quarters to four‐fifths of the circumference via endoscopic submucosal dissection. Postoperative complications associated with ARMS and ARMA include postoperative bleeding in 5% of cases and transient strictures in approximately 13%, primarily due to the healing process of artificial ulcers, which typically takes three to four weeks.^12^, ^14^, ^20^, ^21^, ^22^

To address these limitations, we have successfully integrated ARM‐P and ARMV into a novel technique, anti‐reflux mucoplasty with valve (ARM‐P/V).^24^ This approach incorporates the valvuloplasty element of ARMV while leveraging the closure technique of ARM‐P to reduce adverse events. This pilot study aims to evaluate the safety, feasibility, and efficacy of ARM‐P/V.

## MATERIALS AND METHODS

### Study design

This retrospective investigation analyzed data from a prospectively maintained database of patients who underwent ARM‐P/V treatment for proton pump inhibitor (PPI)‐refractory or PPI‐dependent GERD at Showa University Koto Toyosu Hospital, Tokyo, Japan, between April and August 2024. Data was obtained from electronic medical records and patient‐completed questionnaires. The Institutional Review Board of Showa University approved the study (Approval number: 2024‐161‐A).

### Primary and secondary outcome

The primary objectives of this study were to evaluate improvements in symptom severity and quality of life changes through validated questionnaires, including the GERD‐Health Related Quality of Life Questionnaire (GERD‐HRQL),^25^ the GERD Questionnaire (GerdQ),^26^ and the Frequency Scale for the Symptoms of GERD (FSSG).^27^ Pre‐ and post‐treatment scores were compared, and the proportion of patients discontinuing PPI therapy was analyzed. Questionnaires were distributed during outpatient visits, with patients completing them independently and returning them to the reception desk. Pre‐treatment scores were obtained while patients were still on PPI therapy, and post‐treatment scores were collected at the first outpatient visit, approximately 2 months after discharge. If the questionnaires were not submitted, Kazuki Yamamoto followed up by phone to either request their submission or directly record the responses. The study also included an assessment of esophagogastric junction morphology using Hill's Classification of the gastroesophageal flap valve (GEFV).^28^ Additionally, intragastric pressure (IGP) was evaluated using the Endoscopic Pressure Study Integrated System (EPSIS), which continuously measures IGP during gastric insufflation through esophagogastroduodenoscopy (EGD).^29^, ^30^, ^31^, ^32^ EPSIS parameters, previously validated as reliable for assessing EARTh outcomes,^32^ were compared before and after treatment.

### Patient selection

Eligible participants experienced reflux symptoms at least twice weekly despite a minimum of 6 months of PPI or potassium‐competitive acid blocker (P‐CAB) therapy, indicating drug‐refractory or drug‐dependent GERD. High‐resolution manometry (HRM; Star Medical Inc., Tokyo, Japan) was used to exclude primary esophageal motility disorders, though patients with ineffective motility as defined by the Chicago Classification^33^, ^34^ were included. Diagnostic evaluations included EGD, barium esophagography, and 24‐h impedance‐pH (MII‐pH) monitoring (ZepHr; Sandhill Scientific Inc.), with acid suppression therapy discontinued at least seven days prior. Esophageal biopsies were conducted for suspected eosinophilic esophagitis.^35^, ^36^ GERD diagnosis followed the Lyon Consensus 2.0 criteria,^37^ considering acid exposure time, total reflux episodes, mean nocturnal baseline impedance (MNBI), and endoscopic findings. Patients with non‐erosive GERD and without pathological acid reflux (acid exposure time <4.0%, total reflux episodes <40/day, MNBI >2500 Ω) or with negative symptom index ^38^ or symptom association probability^39^ results (<50% and <95%, respectively) were excluded. Other exclusions included significant comorbidities, or contraindications to endoscopic procedures.

### Data collection

Patient demographics and clinical data, such as age, gender, body mass index (BMI), GERD symptom duration, American Society of Anesthesiologists Physical Status (ASA‐PS) classification, and the use of antiplatelet or anticoagulant medications, were obtained from electronic medical records. Additional parameters included GERD classifications, such as the Los Angeles (LA) classification, Hill's Classification, and MII‐pH reflux data, as well as information on prior GERD treatments. Perioperative details were also collected, covering procedure duration, postoperative hospital stay, closure techniques, and any adverse events. The procedure duration was defined as the time elapsed from endoscope insertion to the deployment of the final clip. Adverse events were categorized using the Clavien‐Dindo classification system,^40^ a widely recognized method for objectively and consistently grading complications. Pre‐ and post‐treatment outcomes were assessed using validated patient‐reported tools, including the GERD‐HRQL, GerdQ, and FSSG questionnaires. In Japan, the LA classification incorporates grades M (minimal change),^41^ with grades N and M classified as non‐erosive GERD. Endoscopic evaluations, including assessments based on the LA and Hill's classifications, were independently reviewed and confirmed by Kazuki Yamamoto and Ippei Tanaka, both board‐certified members of the Japanese Society of Gastrointestinal Endoscopy.

### Statistical analysis

Continuous variables were expressed as mean values accompanied by their standard deviations (SDs), while categorical variables were summarized as frequencies with their respective percentages. To compare data collected before and after the ARM‐P/V procedure, the Wilcoxon matched‐pairs signed‐ranks test was utilized. All statistical analyses were carried out using Stata software (version 16.1; STATA Corp.), with a significance threshold set at a *p*‐value of <0.05.

### ARM‐P/V procedure

**FIGURE 1:**
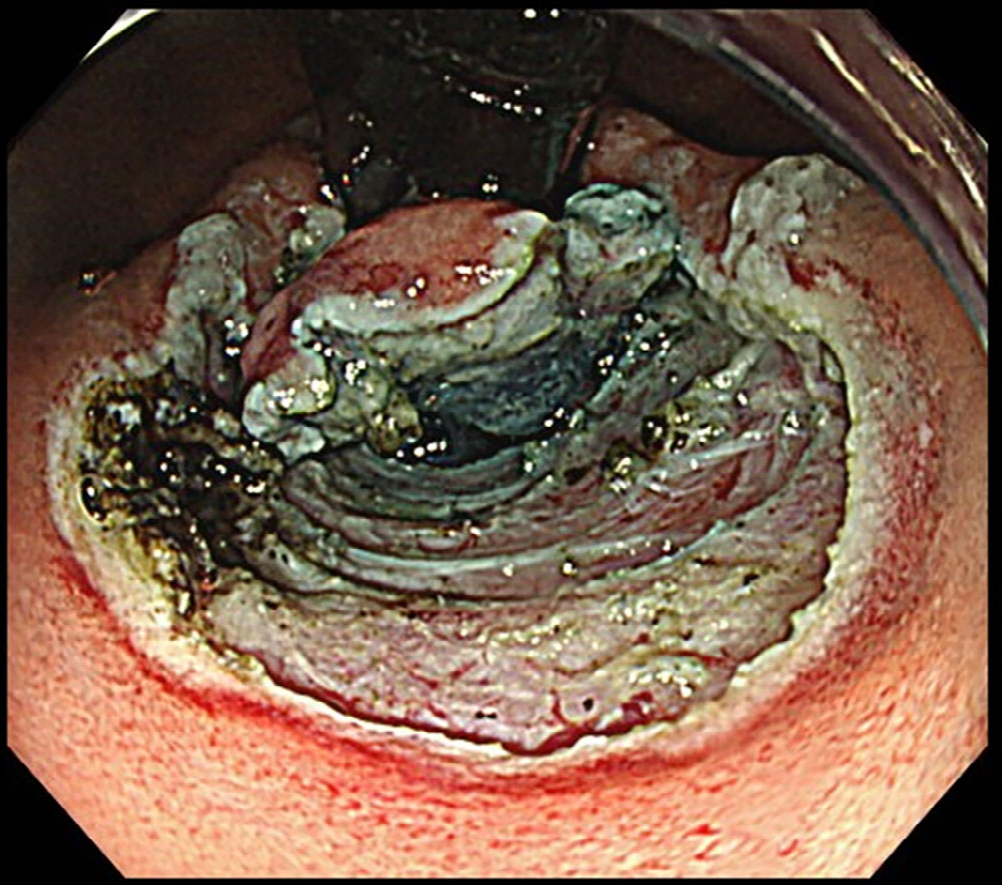
Approximately one‐third of the cardia along the lesser curvature was dissected using the endoscopic submucosal dissection technique to preserve both the mucosal and submucosal layers.

**FIGURE 2:**
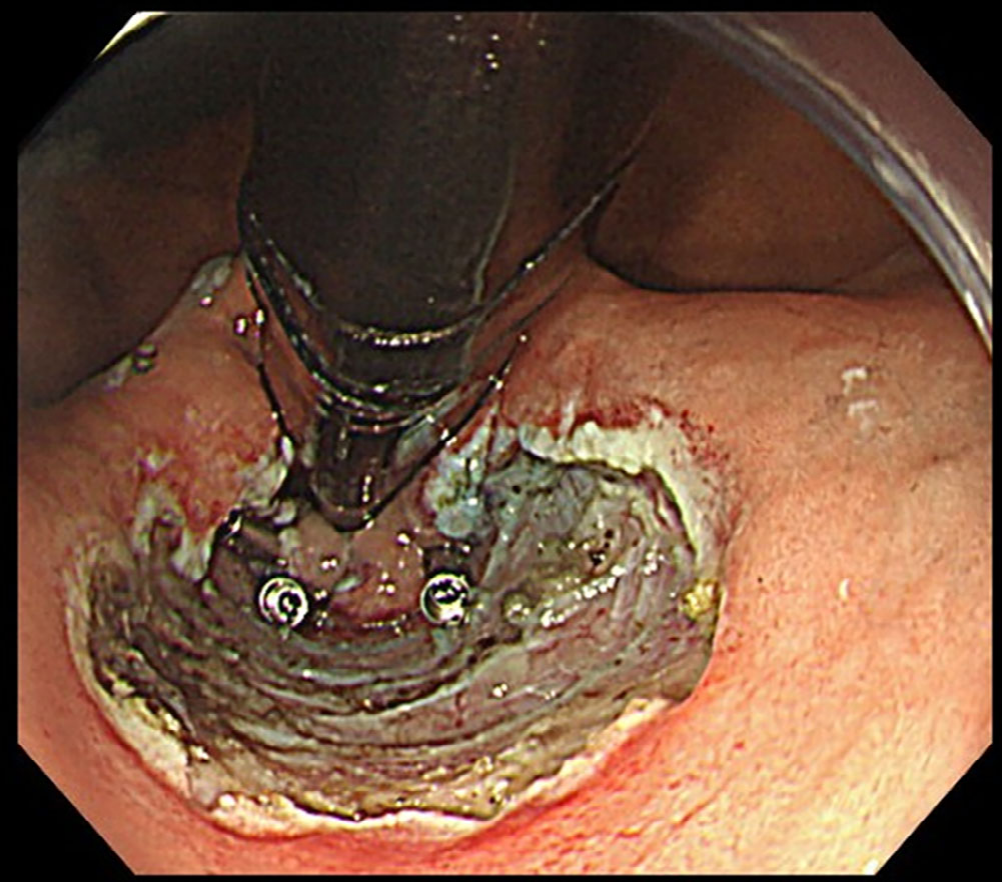
Three reopenable endoclips (SureClip; Microtech) were used to secure the mucosal edge to the muscle layer, maintaining flap elevation.

**FIGURE 3:**
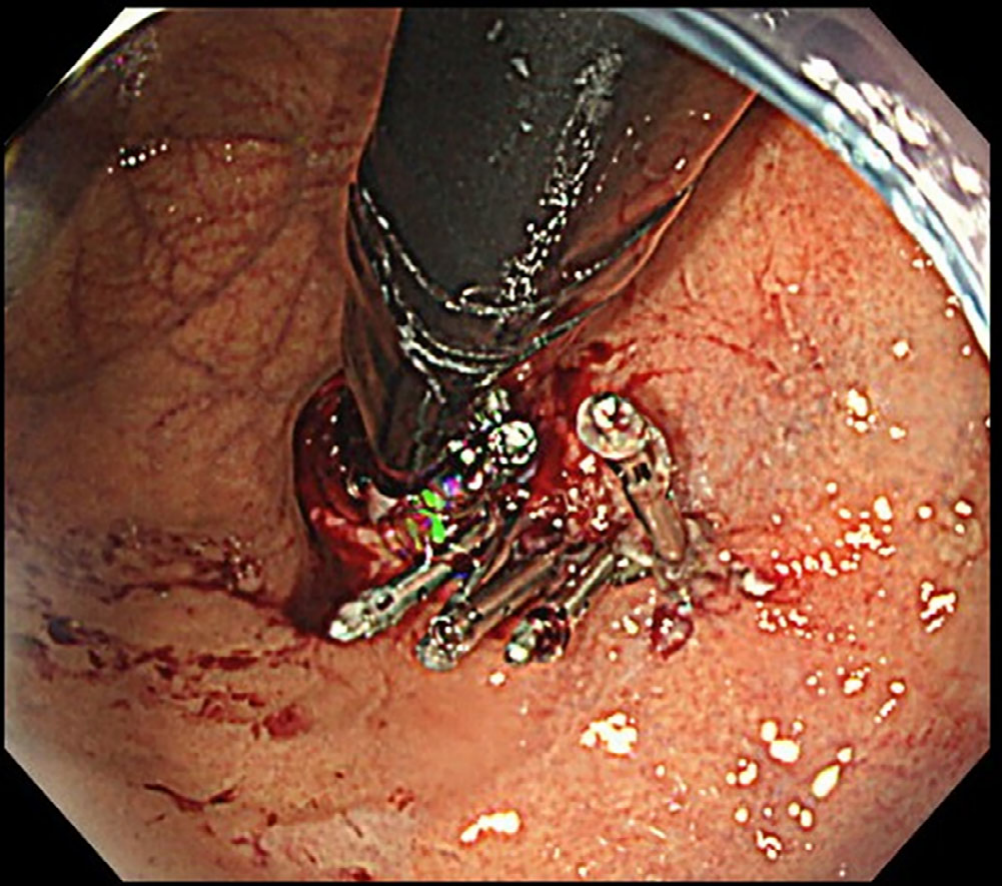
Mucosal defects were closed with anchor‐pronged clips (MANTIS; Boston Scientific).

Under general anesthesia with endotracheal intubation, all procedures were performed by a single operator (Haruhiro Inoue) using a therapeutic endoscope (H290T; Olympus)^24^ with a super‐soft hood (Space Adjuster; TOP)^42^ attached, an endoscopic snare (Smart Snare Hex25; TOP Corporation or Snare Master 25 mm; Olympus), and silk thread (NA11SW; NescoSuture) for angulation control.^43^ For endoscopic submucosal dissection, an electrosurgical unit (VIO3; ERBE Elektromedizin GmbH) was employed with Endocut I current settings of 1‐3‐3. Mucosal incisions were made along approximately one‐third of the lesser curvature circumference using a Triangle Tip Knife J (TTJ; Olympus) after saline with indigo carmine was injected. Submucosal dissection was continued until a double flap of semi‐free mucosa was formed (Figure 1). Two to three reopenable endoclips (SureClip; Microtech) secured the mucosal edge to the muscle layer to maintain flap elevation (Figure 2). Mucosal defects were closed using established techniques, including loop clips (Loop‐9),^44^ anchor pronged clips (MANTIS; Boston Scientific; Figure 3),^45^ and endoscopic hand suturing with a needle holder (FG‐260Q; Olympus), and 3‐0 V‐Loc sutures (VLOCN0804; Medtronic).^46^ The choice of closure method was solely at the operator's discretion.

### Postoperative management and follow‐up

**FIGURE 4:**
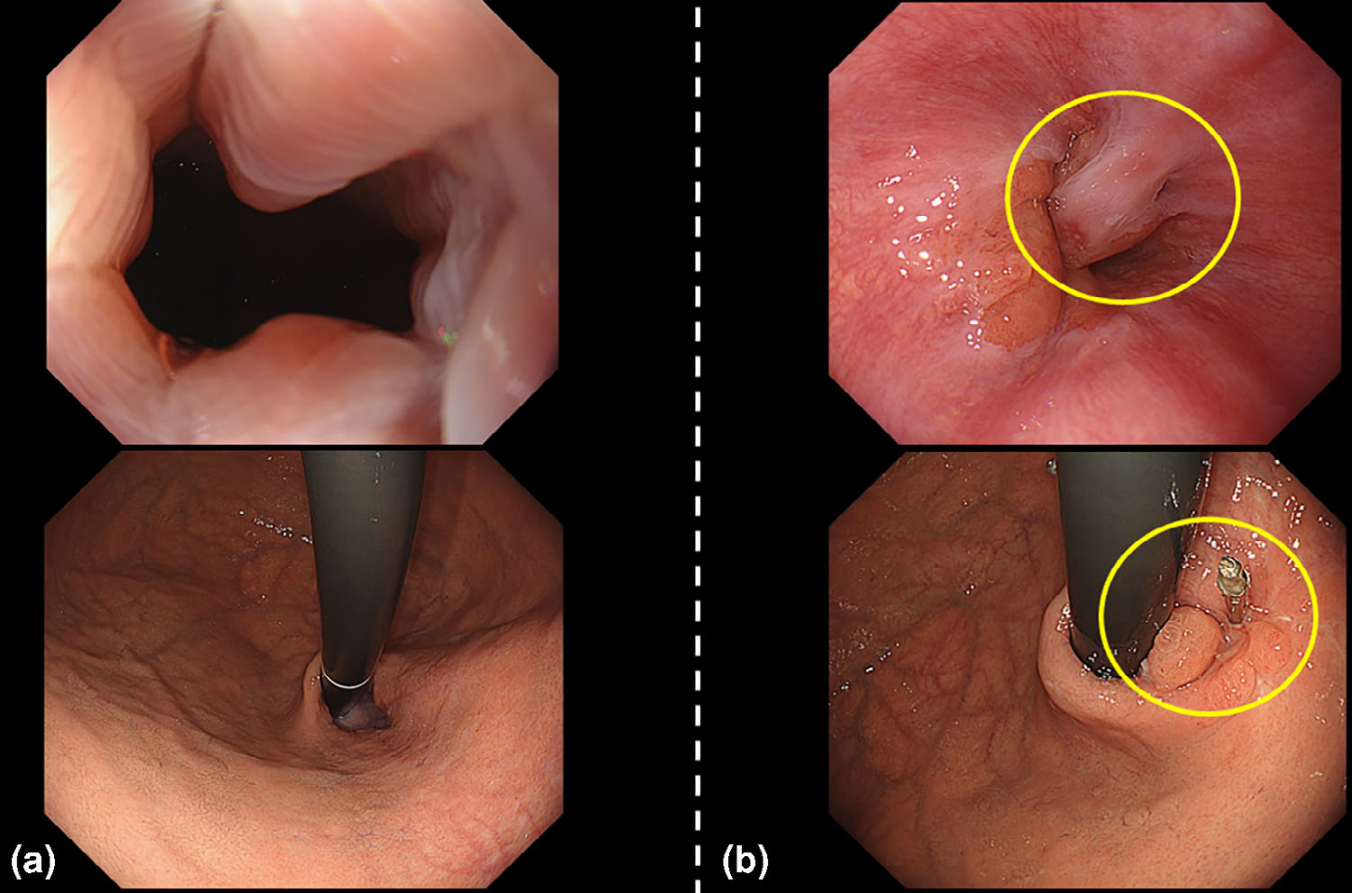
(a) Endoscopic image before anti‐reflux mucoplasty with valve (ARM‐P/V). (b) The postoperative endoscopic follow‐up revealed valve formation with remodeling of the gastroesophageal flap valve and a reduction in hernia prominence. The yellow circle highlights the newly formed valve.

Postoperative endoscopy (GIF‐1200N; Olympus) was performed on the first and fourth or fifth postoperative days to monitor for complications. Symptoms such as chest pain, dyspnea, hematemesis, melena, and abdominal pain were closely observed. Vital signs were checked every 6 h, and blood tests were performed on the first postoperative day. If no complications were identified, patients transitioned to a clear liquid diet. After achieving stable oral intake and completing follow‐up endoscopy, patients were discharged. Follow‐up endoscopy was scheduled approximately 1–3 months post‐procedure, during which questionnaire responses and EPSIS evaluations were conducted. The postoperative endoscopic follow‐up (GIF‐XZ1200; Olympus) showed valve formation with remodeling of the GEFV and reduced hernia prominence (Figure 4).

### EPSIS procedure

EPSIS was conducted before and after treatment using high‐definition endoscopes (GIF‐XZ1200; Olympus) under intravenous propofol sedation and continuous CO_2_ insufflation, following the same methodology as previously reported.^29^, ^30^, ^31^, ^32^ A disposable irrigation tube (AF‐WT; Forte Grow Medical Corp.) connected to a pressure measurement device (TR‐W550, TR‐TH08, AP‐C35; Keyence) recorded IGP parameters. Basal IGP (IGP‐Basal), maximum IGP (IGP‐Max) at the timing of belching, and insufflation time were measured. Pressure differences between IGP‐Basal and IGP‐Max were calculated, with a maximum threshold of 25 mmHg to avoid tissue injury. The waveform gradient was determined by dividing the pressure difference by the insufflation time.

## RESULTS

Eighteen patients who underwent the ARM‐P/V procedure were included in this study. The mean age was 55.4 years (SD 14.8). Baseline patient characteristics are summarized in Table 1. Of the participants, 55.6% (10/18) were male, with a mean GERD symptom duration of 7.0 years (SD 7.6). Half of the patients were classified as ASA I, while the remaining half were ASA II. Only one patient was on regular antiplatelet therapy. Non‐erosive reflux disease was diagnosed in 72.2% (13/18) of participants, with most patients classified as Hill's grade II (27.8%, 5/18) or grade III (66.7%, 12/18). None of the patients had undergone prior interventions for GERD. According to the Lyon Consensus 2.0 criteria, three patients met the criteria for conclusive evidence of GERD, eight had borderline or adjunctive evidence for GERD, and seven had borderline or adjunctive evidence for GERD with high symptom index and symptom association probability.

**TABLE 1:** Comprehensive overview of the baseline characteristics of patients included in this study.

**Patients’ demographics (*n* = 18)**	
Age, mean (SD), years	55.4 (14.8)
Male, *n* (%)	10 (55.6)
BMI, mean (SD), kg/m^2^	22.3 (3.5)
Duration of GERD symptoms, mean (SD), years	7.0 (7.6)
ASA‐PS score, *n* (%)	
Class I	9 (50.0)
Class II	9 (50.0)
Antiplatelet or anticoagulation use, *n* (%)	1 (5.6)
**Endoscopic findings**	
**Esophagitis (LA classification)**	
None or Grade M, *n* (%)	13 (72.2)
Grade A, *n* (%)	3 (16.7)
Grade B, *n* (%)	2 (11.1)
**Hill's Classification of Gastro‐esophageal Flap Valve**	
Grade I, *n* (%)	0 (0.0)
Grade II, *n* (%)	5 (27.8)
Grade III, *n* (%)	12 (66.7)
Grade IV, *n* (%)	1 (5.6)
**24‐h impedance‐pH monitoring**	
Acid exposure time, mean (SD), %	2.2 (2.1)
Total number of reflux events, mean (SD), times	50.5 (27.0)
Number of acid reflux events, mean (SD), times	29.4 (22.0)
Number of non‐acid reflux events, mean (SD), times	21.1 (15.7)
**Previous intervention**	
None, *n* (%)	18 (100.0)

Abbreviations: ASA‐PS, American Society of Anesthesiologists physical status; BMI, body mass index; GERD, gastroesophageal reflux disease; LA, Los Angeles; SD, standard deviation.

The average procedure duration was 77 min (SD 28.3; Table 2), and the mean hospital stay was 4 days (SD 1.5). The closure methods primarily involved only anchor‐pronged clips in 77.8% (14/18) of cases, while a combination of Loop‐9 and anchor‐pronged clips was used in 16.7% (3/18), and endoscopic hand suturing was utilized in one case. No severe complications exceeding Grade 3 on the Clavien‐Dindo classification scale were reported. Most adverse events were Grade 0 (88.9%, 16/18), while two cases were classified as Grade 2 due to antibiotic administration. There were no reports of delayed bleeding or dysphagia requiring balloon dilation after discharge.

**TABLE 2:** Overview of perioperative outcomes in the patients included in this study.

**Perioperative results (*n* = 18)**	
General anesthesia, n (%)	18 (100.0)
Total operation time, mean (SD), minutes	77.7 (28.3)
Postoperative stay, mean (SD), days	4 (1.5)
**Closure method**	
Anchor‐pronged clips only	14 (77.8)
Loop‐9 technique with anchor pronged clips, *n* (%)	3 (16.7)
Endoscopic hand suturing	1 (5.6)
**Adverse events**	
Clavien‐Dindo classification	
Grade 0, *n* (%)	16 (88.9)
Grade 1, *n* (%)	0 (0.0)
Grade 2, *n* (%)	2 (11.1)
Grade 3, *n* (%)	0 (0.0)
Delayed bleeding, *n* (%)	0 (0.0)
Dysphasia requiring dilation, *n* (%)	0 (0.0)

SD, standard deviation.

**FIGURE 5:**
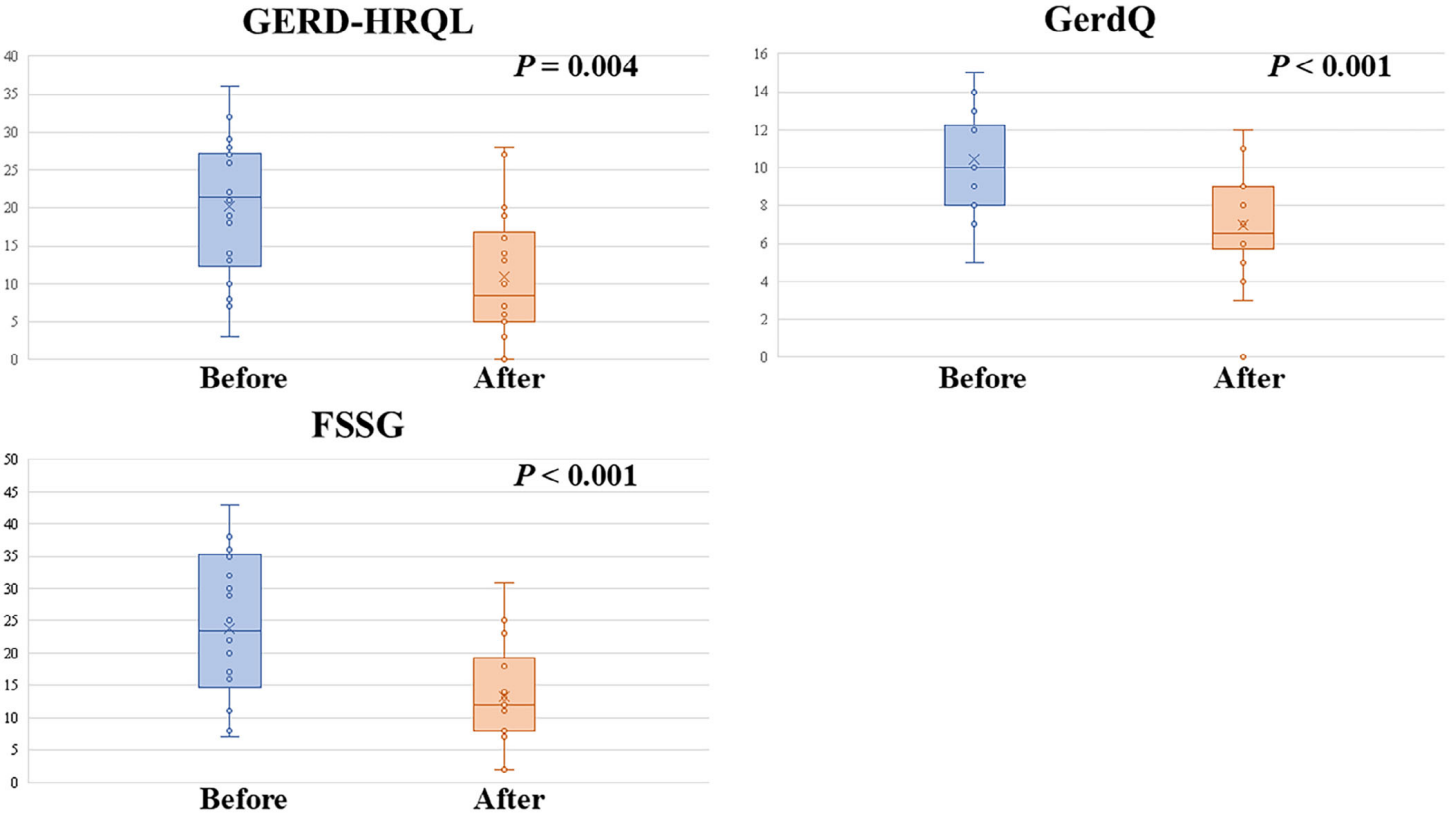
Patient‐reported outcomes, measured by the GERD‐Health Related Quality of Life Questionnaire (GERD‐HRQL), GERD Questionnaire (GERDQ), and Frequency Scale for the Symptoms of GERD (FSSG) score, demonstrated significant improvement following endoscopic anti‐reflux therapy.

The mean follow‐up period was 60.9 days (SD 33.0) (Table 3), during which endoscopic evaluations confirmed complete healing of ARM‐P/V‐related scars. Following the procedure, 55.6% (10/18) of patients successfully discontinued PPI therapy, and 16.7% (3/18) reduced their dosage or switched to on‐demand use. Significant improvements in Hill's classification were observed, with a decrease from a mean of 2.8 (SD 0.5) to 1.2 (SD 0.5; *p* < 0.001). Patient‐reported outcomes showed substantial improvement across all measures (Figure 5). The GERD‐HRQL score dropped from 20.3 (SD 9.4) pre‐procedure to 10.9 (SD 8.4) post‐procedure (*p* = 0.004). Similarly, the GerdQ score improved from 10.4 (SD 2.7) to 6.9 (SD 2.9; *p* < 0.001), and the FSSG score decreased from 24.0 (SD 11.5) to 13.2 (SD 8.0; *p* < 0.001). All patients successfully completed the EPSIS procedure without complications. Postoperative EPSIS assessments revealed a significant increase in mean IGP‐Max (12.3 mmHg pre‐procedure vs. 18.8 mmHg post‐procedure, *p* < 0.001). The mean pressure difference and gradient also showed significant increases, with values rising from 6.4 mmHg to 11.9 mmHg (*p* < 0.001) and from 0.11 to 0.21 mmHg/s (*p* < 0.001), respectively.

**TABLE 3:** Assessment of patient outcomes before and after anti‐reflux mucoplasty with valve (*n* = 18).

**Follow‐up period**	
Post‐procedure to endoscopic follow‐up, mean (SD), days	60.9 (33.0)
**Anti‐reflux medications use after ARM‐P/V**	
Discontinuation, *n* (%)	10 (55.6)
50% dose reduction or on‐demand therapy, *n* (%)	3 (16.7)
Continuation, *n* (%)	5 (27.8)

**Hill's classification of gastro‐esophageal flap valve**			
	**Pre‐ARM‐P/V**	**Post‐ARM‐P/V**	***p*‐value**
Grade I, *n* (%)	0 (0.0)	16 (88.9)	< 0.001
Grade II, *n* (%)	5 (27.8)	1 (5.6)	
Grade III, *n* (%)	12 (66.7)	1 (5.6)	
Grade IV, *n* (%)	1 (5.6)	0 (0.0)	

**EPSIS parameters**			
	**Pre‐ARM‐P/V**	**Post‐ARM‐P/V**	***p*‐value**
Maximum IGP, mean, (SD), mmHg	12.3 (4.3)	18.8 (3.0)	<0.001
Pressure difference, mean, (SD), mmHg	6.4 (4.9)	11.9 (3.5)	<0.001
Pressure gradient, mean, (SD), mmHg/s	0.11 (0.08)	0.21 (0.08)	<0.001

Abbreviations: ARM‐P/V, anti‐reflux mucoplasty with valve; EPSIS, Endoscopic Pressure Study Integrated System; IGP, intragastric pressure; SD, standard deviation.

## DISCUSSION

This pilot study highlights promising outcomes for patients with PPI‐refractory or PPI‐dependent GERD who underwent ARM‐P/V. The procedure was well‐tolerated and led to substantial symptom improvement and reduction in acid reflux.

Patients experienced marked symptom relief, as evidenced by the significant number (72.2%, 13/18) who either discontinued PPI therapy, reduced their dosage, or transitioned to on‐demand use within a mean follow‐up period of approximately 3 months. Patient‐reported outcomes across all metrics demonstrated significant improvements after ARM‐P/V, consistent with prior studies on ARMS,^12^, ^13^ ARMA,^14^, ^15^ and ARM‐P,^16^, ^17^ procedures, as well as reviews and meta‐analyses of these techniques.^20^, ^21^, ^22^ While this study focuses on short‐term outcomes and long‐term efficacy remains unexplored, the sustained benefits observed in previous studies of ARMA and ARMS, lasting up to 3^19^ and 5 years,^18^ respectively, suggest that similar long‐term results could be anticipated with ARM‐P/V.

Notable improvements in Hill's Classification of the GEFV were observed, addressing a recognized factor in refractory GERD.^28^, ^36^ EPSIS findings corroborated these outcomes, with significant post‐procedure enhancements in parameters such as IGP‐Max, pressure differences, and pressure gradients. Post‐treatment EPSIS waveforms displayed marked improvements, reflecting effective LES remodeling due to ARM‐P/V. Although MII‐pH monitoring remains the gold standard for reflux evaluation, EPSIS has demonstrated reliability through its correlations with MII‐pH monitoring,^29^ endoscopic findings,^30^ HRM,^31^ and also post‐treatment outcomes after EARTh.^32^ This study confirmed consistent results in lower esophageal sphincter remodeling and EPSIS parameters, even with novel techniques like ARM‐P/V.

During the follow‐up period, no severe complications exceeding Grade 3 on the Clavien‐Dindo classification scale were observed. The majority of cases were classified as Grade 0 (88.9%, 16/18), while two cases were Grade 2 due to fever with the prophylactic administration of intravenous antibiotics. However, both patients were discharged without the need for additional endoscopic or surgical interventions, similar to the other participants. Importantly, there were no cases of delayed bleeding or dysphagia necessitating balloon dilation. The bleeding risk associated with ARMS and ARMA, estimated at approximately 5%, is higher in patients on antiplatelet medications.^12^, ^14^, ^20^, ^21^, ^22^ This increased risk is often linked to open ulcers that can persist for about three weeks post‐procedure and may lead to delayed bleeding. In ARM‐P/V, the smaller dissection area, approximately one‐third of the mucosa, combined with prompt closure, contrasts with the three‐quarters to four‐fifths mucosal resection required in ARMS, ARMA, and ARMV procedures.^12^, ^13^, ^15^, ^23^ This reduced resection area likely plays a key role in minimizing bleeding risk. Regarding dysphagia, the incidence with ARMS or ARMA is reported to range from 7% to 13%,^12^, ^14^, ^20^, ^21^, ^22^ yet no patients in this study experienced dysphagia or required balloon dilation following ARM‐P/V. This outcome is consistent with findings from the ARM‐P study, likely due to the prompt and effective defect closure, which minimizes the variability commonly observed with ARMS and ARMA procedures.

It is important to acknowledge the limitations of this study, which include its pilot design with a single‐arm approach, a small sample size, and a short follow‐up period. To draw more robust conclusions, larger randomized controlled trials are necessary. Additionally, the lack of post‐procedure MII‐pH monitoring data constrains the ability to comprehensively evaluate treatment efficacy. However, prior research has already demonstrated the effectiveness of EPSIS in assessing post‐treatment outcomes after EARTh,^32^ with this technique showing potential to enhance GERD treatment success rates by approximately 60–70%. These findings build on the established efficacy of ARMS and ARMA procedures.^20^, ^21^, ^22^ Moreover, regarding the questionnaires, when not returned, responses were obtained by phone through direct inquiry by the investigator, which may introduce response bias. However, since only one patient required direct involvement, the potential impact of this bias is considered minor. Finally, while we previously demonstrated the efficacy of ARM‐P in endoscopic anti‐reflux therapy,^16^ this pilot study highlights the potential for valvuloplasty to further reinforce the anti‐reflux mechanism. Nevertheless, the long‐term durability of the mucosal valve created in ARM‐P/V and its superiority over ARM‐P remain uncertain. Future randomized controlled trials and multicenter studies are necessary to provide conclusive evidence.

## CONCLUSION

In conclusion, ARM‐P/V is a safe, technically feasible, and effective short‐term treatment for GERD. As an advancement of ARMV, it represents a promising alternative to existing techniques. However, further studies are required to assess its long‐term efficacy and to compare its benefits with those of established methods.

## CONFLICT OF INTEREST STATEMENT

Haruhiro Inoue is an advisor of Olympus Corporation and Top Corporation. The authors declare no conflicts of interest.

## ETHICS STATEMENT

The study received approval by the Institutional Review Board of the Showa University (Approval number: 2024‐161‐A).

## PATIENT CONSENT STATEMENT

Not applicable.

## CLINICAL TRIAL REGISTRATION

N/A.

## References

[1] Ustaoglu A , Nguyen A , Spechler S , Sifrim D , Souza R , Woodland P . Mucosal pathogenesis in gastro‐esophageal reflux disease. Neurogastroenterol Motil 2020; 32: e14022. 33118247 10.1111/nmo.14022

[2] Dean BB , Gano AD Jr , Knight K , Ofman JJ , Fass R . Effectiveness of proton pump inhibitors in nonerosive reflux disease. Clin Gastroenterol Hepatol 2004; 2: 656–64. 15290657 10.1016/s1542-3565(04)00288-5

[3] Zornig C , Strate U , Fibbe C , Emmermann A , Layer P . Nissen vs toupet laparoscopic fundoplication. Surg Endosc 2002; 16: 758–66. 11997817 10.1007/s00464-001-9092-8

[4] Toupet A . [Technic of esophago‐gastroplasty with phrenogastropexy used in radical treatment of hiatal hernias as a supplement to Heller's operation in cardiospasms]. Mem Acad Chir 1963; 89: 384–9. 13993831

[5] Nissen R . [A simple operation for control of reflux esophagitis]. Schweiz Med Wochenschr 1956; 86: 590–2. 13337262

[6] Kalapala R , Karyampudi A , Nabi Z *et al.* Endoscopic full‐thickness plication for the treatment of PPI‐dependent GERD: Results from a randomised, sham controlled trial. Gut 2022; 71: 686–94. 33849942 10.1136/gutjnl-2020-321811 PMC8921577

[7] Testoni S , Hassan C , Mazzoleni G *et al.* Long‐term outcomes of transoral incisionless fundoplication for gastro‐esophageal reflux disease: Systematic‐review and meta‐analysis. Endosc Int Open 2021; 9: E239–46. 33553587 10.1055/a-1322-2209 PMC7857958

[8] Rodriguez de Santiago E , Albeniz E , Estremera‐Arevalo F , Teruel Sanchez‐Vegazo C , Lorenzo‐Zuniga V . Endoscopic anti‐reflux therapy for gastroesophageal reflux disease. World J Gastroenterol 2021; 27: 6601–14. 34754155 10.3748/wjg.v27.i39.6601 PMC8554403

[9] Aziz AM , El‐Khayat HR , Sadek A *et al.* A prospective randomized trial of sham, single‐dose stretta, and double‐dose stretta for the treatment of gastroesophageal reflux disease. Surg Endosc 2010; 24: 818–25. 19730952 10.1007/s00464-009-0671-4

[10] Monino L , Gonzalez JM , Vitton V , Barthet M . Antireflux mucosectomy band in treatment of refractory gastroesophageal reflux disease: A pilot study for safety, feasibility and symptom control. Endosc Int Open 2020; 8: E147–54. 32010747 10.1055/a-1038-4012 PMC6976317

[11] Debourdeau A , Vitton V , Monino L , Barthet M , Gonzalez JM . Antireflux mucosectomy band (ARM‐b) in treatment of refractory gastroesophageal reflux disease after bariatric surgery. Obes Surg 2020; 30: 4654–8. 32676843 10.1007/s11695-020-04753-4

[12] Sumi K , Inoue H , Kobayashi Y *et al.* Endoscopic treatment of proton pump inhibitor‐refractory gastroesophageal reflux disease with anti‐reflux mucosectomy: Experience of 109 cases. Dig Endosc 2021; 33: 347–54. 32415898 10.1111/den.13727

[13] Inoue H , Ito H , Ikeda H *et al.* Anti‐reflux mucosectomy for gastroesophageal reflux disease in the absence of hiatus hernia: A pilot study. Ann Gastroenterol 2014; 27: 346–51. 25330784 PMC4188931

[14] Shimamura Y , Inoue H , Tanabe M *et al.* Clinical outcomes of anti‐reflux mucosal ablation for gastroesophageal reflux disease: An international bi‐institutional study. J Gastroenterol Hepatol 2024; 39: 149–56. 37787176 10.1111/jgh.16370

[15] Inoue H , Tanabe M , de Santiago ER *et al.* Anti‐reflux mucosal ablation (ARMA) as a new treatment for gastroesophageal reflux refractory to proton pump inhibitors: A pilot study. Endosc Int Open 2020; 8: E133–8. 32010745 10.1055/a-1031-9436 PMC6976329

[16] Inoue H , Yamamoto K , Shimamura Y *et al.* Pilot study on anti‐reflux mucoplasty: Advancing endoscopic anti‐reflux therapy for gastroesophageal reflux disease. Dig Endosc 2024; 36: 690–8. 37899073 10.1111/den.14711 PMC12108231

[17] Inoue H , Yamamoto K , Navarro MJ *et al.* Antireflux mucoplasty, an evolution of endoscopic antireflux therapy for refractory GERD. VideoGIE 2023; 8: 435–40. 38026716 10.1016/j.vgie.2023.07.003 PMC10665536

[18] Sumi K , Inoue H , Ando R *et al.* Long‐term efficacy of antireflux mucosectomy in patients with refractory gastroesophageal reflux disease. Dig Endosc 2024; 36: 305–13. 37332095 10.1111/den.14617

[19] Hernandez Mondragon OV , Zamarripa Mottu RA , Garcia Contreras LF *et al.* Clinical feasibility of a new antireflux ablation therapy on gastroesophageal reflux disease (with video). Gastrointest Endosc 2020; 92: 1190–201. 32343977 10.1016/j.gie.2020.04.046

[20] Yeh JH , Lee CT , Hsu MH *et al.* Antireflux mucosal intervention (ARMI) procedures for refractory gastroesophageal reflux disease: A systematic review and meta‐analysis. Therap Adv Gastroenterol 2022; 15: 17562848221094959. 10.1177/17562848221094959 PMC9058334 35509424

[21] Garg R , Mohammed A , Singh A *et al.* Anti‐reflux mucosectomy for refractory gastroesophageal reflux disease: A systematic review and meta‐analysis. Endosc Int Open 2022; 10: E854–E64. 35692929 10.1055/a-1802-0220 PMC9187426

[22] Rodriguez de Santiago E , Sanchez‐Vegazo CT , Penas B *et al.* Antireflux mucosectomy (ARMS) and antireflux mucosal ablation (ARMA) for gastroesophageal reflux disease: A systematic review and meta‐analysis. Endosc Int Open 2021; 9: E1740–51. 34790538 10.1055/a-1552-3239 PMC8589565

[23] Lu J , Chen F , Lv X *et al.* Endoscopic construction of an antireflux mucosal barrier for the treatment of GERD: A pilot study (with video). Gastrointest Endosc 2023; 98: 1017–22. 37660832 10.1016/j.gie.2023.08.017

[24] Inoue H , Yamamoto K , Tanaka I *et al.* Introducing antireflux mucoplasty with valve: A novel endoscopic treatment for GERD. VideoGIE 2024; 9: 463–7. 39534560 10.1016/j.vgie.2024.06.009 PMC11551455

[25] Velanovich V . The development of the GERD‐HRQL symptom severity instrument. Dis Esophagus 2007; 20: 130–4. 17439596 10.1111/j.1442-2050.2007.00658.x

[26] Jones R , Junghard O , Dent J *et al.* Development of the GERDQ, a tool for the diagnosis and management of gastro‐oesophageal reflux disease in primary care. Aliment Pharmacol Ther 2009; 30: 1030–8. 19737151 10.1111/j.1365-2036.2009.04142.x

[27] Kusano M , Shimoyama Y , Sugimoto S *et al.* Development and evaluation of FSSG: Frequency scale for the symptoms of GERD. J Gastroenterol 2004; 39: 888–91. 15565409 10.1007/s00535-004-1417-7

[28] Hill LD , Kozarek RA , Kraemer SJ *et al.* The gastroesophageal flap valve: In vitro and in vivo observations. Gastrointest Endosc 1996; 44: 541–7. 8934159 10.1016/s0016-5107(96)70006-8

[29] Inoue H , Shimamura Y , Rodriguez de Santiago E *et al.* Diagnostic performance of the endoscopic pressure study integrated system (EPSIS): A novel diagnostic tool for gastroesophageal reflux disease. Endoscopy 2019; 51: 759–62. 31216578 10.1055/a-0938-2777

[30] Iwaya Y , Inoue H , Rodriguez de Santiago E *et al.* Endoscopic pressure study integrated system reflects gastroesophageal junction competence in patients with erosive esophagitis and Barrett's esophagus. Dig Endosc 2020; 32: 1050–6. 32012362 10.1111/den.13644

[31] Fujiyoshi Y , Inoue H , Shimamura Y *et al.* Association between endoscopic pressure study integrated system (EPSIS) and high‐resolution manometry. Endosc Int Open 2022; 10: E762–8. 35692910 10.1055/a-1790-6141 PMC9187419

[32] Yamamoto K , Inoue H , Tanaka I *et al.* The utility of endoscopic pressure study integrated system for gastroesophageal reflux disease after endoscopic anti‐reflux therapy. DEN Open (in press) 2025 DOI: 10.1111/den.14989. 39833986

[33] Yadlapati R , Kahrilas PJ , Fox MR *et al.* Esophageal motility disorders on high‐resolution manometry: Chicago classification version 4.0((c)). Neurogastroenterol Motil 2021; 33: e14058. 33373111 10.1111/nmo.14058 PMC8034247

[34] Kahrilas PJ , Bredenoord AJ , Fox M *et al.* The Chicago classification of esophageal motility disorders, v3.0. Neurogastroenterol Motil 2015; 27: 160–74. 25469569 10.1111/nmo.12477 PMC4308501

[35] Muller S , Puhl S , Vieth M , Stolte M . Analysis of symptoms and endoscopic findings in 117 patients with histological diagnoses of eosinophilic esophagitis. Endoscopy 2007; 39: 339–44. 17427070 10.1055/s-2007-966216

[36] Tack J , Pandolfino JE . Pathophysiology of gastroesophageal reflux disease. Gastroenterology 2018; 154: 277–88. 29037470 10.1053/j.gastro.2017.09.047

[37] Gyawali CP , Yadlapati R , Fass R *et al.* Updates to the modern diagnosis of GERD: Lyon consensus 2.0. Gut 2024; 73: 361–71. 37734911 10.1136/gutjnl-2023-330616 PMC10846564

[38] Wiener GJ , Richter JE , Copper JB , Wu WC , Castell DO . The symptom index: A clinically important parameter of ambulatory 24‐hour esophageal pH monitoring. Am J Gastroenterol 1988; 83: 358–61. 3348191

[39] Weusten BL , Roelofs JM , Akkermans LM , Van Berge‐Henegouwen GP , Smout AJ . The symptom‐association probability: An improved method for symptom analysis of 24‐hour esophageal pH data. Gastroenterology 1994; 107: 1741–5. 7958686 10.1016/0016-5085(94)90815-x

[40] Dindo D , Demartines N , Clavien PA . Classification of surgical complications: A new proposal with evaluation in a cohort of 6336 patients and results of a survey. Ann Surg 2004; 240: 205–13. 15273542 10.1097/01.sla.0000133083.54934.ae PMC1360123

[41] Dent J . Endoscopic grading of reflux oesophagitis: The past, present and future. Best Pract Res Clin Gastroenterol 2008; 22: 585–99. 18656818 10.1016/j.bpg.2008.01.002

[42] Fujiyoshi Y , Shimamura Y , Mosko JD , Inoue H . Endoscopic submucosal dissection using a new super‐soft hood and the multipoint traction technique. VideoGIE 2020; 5: 274–7. 32642609 10.1016/j.vgie.2020.03.010 PMC7332761

[43] Inoue H , Navarro MJ , Ushikubo K *et al.* Handmade snare‐assisted endoscope tip‐bending angulation booster. VideoGIE 2024; 9: 511–5. 39698406 10.1016/j.vgie.2024.08.006 PMC11652100

[44] Inoue H , Tanabe M , Shimamura Y *et al.* A novel endoscopic purse‐string suture technique, “loop 9”, for gastrointestinal defect closure: A pilot study. Endoscopy 2022; 54: 158–62. 33472242 10.1055/a-1364-4160

[45] Yamamoto K , Inoue H , Tanaka I *et al.* Closure in antireflux mucoplasty using anchor prong clips: Dead space‐eliminating technique. VideoGIE 2024; 9: 303–8. 39070685 10.1016/j.vgie.2024.03.010 PMC11281917

[46] Ushikubo K , Inoue H , Yamamoto K *et al.* Enhancing closure efficacy in antireflux mucoplasty through endoscopic hand‐suturing technique. VideoGIE 2024; 9: 259–61. 38887733 10.1016/j.vgie.2024.03.002 PMC11180374

